# Ceramic Crowns and Sleep Bruxism: First Results from a Randomized Trial

**DOI:** 10.3390/jcm12010273

**Published:** 2022-12-29

**Authors:** Marc Schmitter, Wolfgang Bömicke, Rouven Behnisch, Justo Lorenzo Bermejo, Moritz Waldecker, Peter Rammelsberg, Brigitte Ohlmann

**Affiliations:** 1 Department of Prosthodontics, University of Würzburg, Pleicherwall 2, 97070 Würzburg, Germany; 2 Department of Prosthodontics, University of Heidelberg, Im Neuenheimer Feld 400, 69120 Heidelberg, Germany; 3 Institute of Medical Biometry, University of Heidelberg, Im Neuenheimer Feld 130.3, 69120 Heidelberg, Germany

**Keywords:** bruxism, cad-cam, ceramics, clinical studies/trials, prosthetic dentistry/prosthodontics, clinical outcomes

## Abstract

Background: This randomized clinical trial was conducted to assess whether sleep bruxism (SB) is associated with an increased rate of technical complications (ceramic defects) in lithium disilicate (LiDi) or zirconia (Z) molar single crowns (SCs). Methods: Adult patients were classified as affected or unaffected by SB based on structured questionnaires, clinical signs, and overnight portable electromyography (BruxOff) and block randomized into four groups according to SB status and crown material (LiDi or Z): LiDi-SB (*n* = 29), LiDi-no SB (*n* = 24), Z-SB (*n* = 23), and Z-no SB (*n* = 27). Differences in technical complications (main outcome) and survival and success rates (secondary outcomes) one year after crown cementation were assessed using Fisher’s exact test with significance level α = 0.05. Results: No technical complications occurred. Restoration survival rates were 100% in the LiDi-SB and LiDi-no SB groups, 95.7% in the Z-SB group, and 96.3% in the Z-no SB group (*p* > 0.999). Success rates were 96.6% in the LiDi-SB group, 95.8% in the LiDi-no SB group (*p* > 0.999), 91.3% in the Z-SB group, and 96.3% in the Z-no SB group (*p* ≥ 0.588). Conclusions: With a limited observation time and sample size, no effect of SB on technical complication, survival, and success rates of molar LiDi and Z SCs was detected.

## 1. Introduction

Lithium disilicate ceramic (LiDi) and zirconia ceramic (Z) are popular restorative materials. Based on their mechanical properties, they can be used to fabricate monolithic posterior single crowns (SCs) [1], but they are brittle and prone to tensile stress. Although these ceramic restorations can withstand the forces of normal mastication and function, extreme biting forces, such as those experienced during bruxism, may lead to technical failure [2]. Bruxism is the clenching or grinding of teeth and can occur during the daytime (awake bruxism) or during sleep (sleep bruxism (SB)) [3].

During SB, biting forces can exceed the maximum voluntary bite force that occurs during daytime [4] and can reach more than 800 N in men [5]. Several studies have assessed the impact of SB on complication rates in ceramic restorations [6], but the overall quality of evidence of these studies was very low according to GRADE criteria [6]. Reasons for this low quality included invalid criteria to identify bruxers, small sample sizes, and poor study design (retrospective and/or non-randomized studies) [6].

Polysomnography is the gold standard for SB diagnostics [7], but is expensive, has limited capacity, and disturbs sleep in participants [8], making it a problematic technique for clinical studies [9,10]. To address these problems, portable electromyographic (EMG) devices have been developed to assess SB [11,12,13]. However, EMG devices overestimate the number of SB episodes because they do not differentiate between muscle activation of jaw muscles and mimic muscles [14]. These limitations can be overcome using the BruxOff device (Spes Medica, Genova, Italy), which records the activity of the left and right masseter muscles [11]. The BruxOff device also records heart rate, which is relevant because the autonomic nervous system is activated and the heart rate increases during SB [15]. Recording the heart rate can avoid overestimation of SB by EMG [11]. Based on this, the BruxOff device was used in this study to verify SB in participants with clinical and anamnestic signs of SB.

In the present study, the technical complication rates of SCs made from LiDi or Z in patients with and without SB were compared. The following null hypotheses were tested: (i) the technical complication rates in patients with LiDi SCs with and without SB are equal, and (ii) the technical complication rates in patients with Z SCs with and without SB are equal.

## 2. Materials and Methods

The study was a two-arm, randomized, single-center clinical study of parallel (1:1) groups. It was approved by the local ethics committee and registered at clinicaltrials.gov (NCT03039985).

Patients (≥18 years) with full legal capacity were eligible to participate in the study if they needed a molar SC for a tooth that was vital or sufficiently endodontically treated, was periodontally stable, and had a natural antagonist (with/without restoration). Exclusion criteria were allergies against the materials used in the study, acute neuropsychiatric disorders, hemorrhagic diatheses, a heart pacemaker, pregnancy, breastfeeding, plans to change residency within the next years, lack of compliance, or inadequate dental hygiene.

Patients were screened for eligibility between 2015 and 2019. Study data were collected after the patient gave written informed consent to participate in the study.

SB was diagnosed according to the American Academy of Sleep Medicine criteria [16,17]. The diagnosis was based on (i) self-reporting of SB, (ii) a clinical examination for signs of SB, and (iii) data from the BruxOff device. In this study, participants were classified as having SB or no SB if all three items confirmed this. Patients who did not meet this requirement were excluded from study participation.

The self-report indicated SB if at least one question in each of the two questionnaires was answered with “yes”. The first of the two questionnaires was developed by Paesani et al. [18] and the second was a structured interview developed by Raphael et al. [19]. If the answers to the two questionnaires were conflicting, the patient was excluded.

The following clinical signs of SB were assessed and any one of these indicated SB: (i) abnormal tooth wear, (ii) cheek impressions of teeth, (iii) tongue impressions of teeth, or (iv) asymmetry/hypertrophy of the masseter muscles.

For EMG/ECG, the portable device was worn for five nights (each with a minimum of five hours of recorded sleep). SB was determined by more than two SB episodes per hour in at least one night. The final SB diagnosis was only disclosed to the patient and not to the treating dentist.

Each participant was assigned the ceramic restorative material based on computer-generated, stratified (SB/no SB) block randomization using lots provided by a biostatistician in sealed and consecutively numbered envelopes. The next lot in the sequence was drawn only after the definitive impression had been taken. The lots were handed out by a study nurse who kept them locked. Participants were blinded to the material from which their SC was made.

All participants were treated at the prosthodontics department of a university hospital, and the study SCs (one per participant) were provided by fully-trained dentists (*n* = 8) between 2015 and 2020.

After removing caries and old restorations, the abutment teeth were built up with a dual-polymerizing composite (Rebilda DC white, VOCO, Cuxhaven, Germany). Preparations were made for a minimum ceramic thickness of 1.2 mm occlusally and 1.0 mm axially, with rounded edges, a chamfer finish line, and a total occlusal convergence angle of 6 to 12 degrees.

All SCs were made by three trained dental technicians, and were virtually designed (Zirkonzahn.Modellier, Zirkonzahn, Gais, Italy) as monolithic restorations. LiDi SCs were milled (Zirkonzahn M1/M5, Zirkonzahn) from a wax blank (wax purple 95H10, Zirkonzahn) and pressed (Programat EP 5000, Ivoclar Vivadent, Schaan, Lichtenstein) from LiDi glass ceramics (IPS e.max Press, Ivoclar Vivadent, Schaan, Liechtenstein). Z SCs were milled (Zirkonzahn M1/M5, Zirkonzahn) from pre-sintered 3 mol% yttria-stabilized-tetragonal-zirconia-polycrystal (Prettau Zirconia, Zirkonzahn) and then colored (Colour Liquid Prettau, Zirkonzahn) and sintered (Zirkonofen 700, Zirkonzahn) at 1600 °C. After fitting in the articulator, all SCs were stained and glaze fired (Programat EP 5000, Ivoclar Vivadent; LiDi: IPS e.max Ceram shade/glaze, Ivoclar Vivadent; Z: ICE ZIRKON 3D STAINS, Zirkonzahn). During the clinical fitting, necessary adjustments were made with ceramic-specific diamond rotary instruments (ZR6881.314.016, ZR6390.314.016, ZR8881.315.016, Gebr. Brasseler, Lemgo, Germany) cooled by water. Proximal/occlusal adjustments were polished to a high gloss in the dental laboratory and a second stain and glaze firing was performed if needed.

Study SCs were inspected by an independent investigator and approved for cementation if they were made of the randomly-assigned material, if the entire extaglio surface appeared smooth and highly polished under 3.5× light microscopic magnification, if no ceramic defects were visible, and if the material thickness was equal to or higher than the specified minimum thickness. SCs were adhesively cemented with self-adhesive dual-polymerizing composite cement (RelyX Unicem, 3M Deutschland, Neuss, Germany). Therefore, the intaglio surfaces of LiDi SCs were etched with 5% hydrofluoric acid (Vita Ceramics Etch, Vita Zahnfabrik H. Rauter, Bad Säckingen, Germany) for 20 s and silanized (Monobond Plus, Ivoclar Vivadent). The intaglio surfaces of the Z SCs were abraded with airborne particles of 50 µm alumina at 0.1 MPa. Occlusal corrections were made after cementation and the adjusted areas were polished using ceramic-specific polishers (set no. 4637.000, Gebr. Brasseler). Any occlusal corrections were documented.

Follow-up examinations were conducted one week, six months, and one year after SC cementation. The SCs and the antagonists were examined, and the occlusion was checked (occlusal adjustments were handled and documented as described above). At the six-month and one-year follow-ups, the probing pocket depths were recorded at six sites per tooth, together with the mobility and vitality of the abutment and antagonist teeth.

The primary outcome of this study was the rate of technical complications (frequency, f) of LiDi SCs or Z SCs between participants with and without SB. The following null hypotheses (H_0_) were formulated:

$$H_{0-A}:f_{LIDI-SB}=f_{LIDI-no\;SB}$$

$$H_{0-B}:f_{Z-SB}=f_{Z-no\;SB}$$

Since complication rates in monolithic restorations could not be assumed based on published data when the study was planned, a two-phase adaptive study design was conducted. In the stage I analysis, an interim analysis was performed one year after the first 25 patients per group were treated, during which chi-square tests were used to compare technical complication rates. In the stage II analysis, calculations were based on the *p*-values from the interim and the final analyses, according to the following rules:

Stage I analysis. Calculate chi-square *p*-values to compare f_LiDi-SB_ vs. f_LiDi-no SB_ (p_A_) and f_Z-SB_ vs f_Z-no SB_ (p_B_). Then, p_1_ = min (p_A_, p_B_).

- if p1 ≤ 0.01, terminate the study and conclude that complication rates are different;
- if p1 > 0.20, terminate the study because of low differences between complication rates;
- if 0.01 ≤ p1 ≤ 0.20, continue to stage II with the two groups that show the smallest *p*-value (pA or pB).
  Stage II analysis. Calculate the chi-square *p*-value p_2_ based on the stage II data.
- if p1 × p2 ≤ 0.013: conclude that complication rates in the selected groups are different;
- otherwise: no difference in complication rates.

Secondary outcomes were also compared between patients in the SB and no SB groups and who received LiDi or Z SCs (Fisher’s exact test, α = 0.05). These secondary outcomes were the survival rate (SC in situ and no replacement required) and success rate (SC in situ without any complication affecting the SC and/or the abutment tooth). All calculations were performed using statistical software (IBM SPSS Statistics v 28.0.0.0, IBM, Armonk, New York, USA and R v 4.1.1/2021-08-10).

## 3. Results

### 3.1. Participants

**Figure 1:**
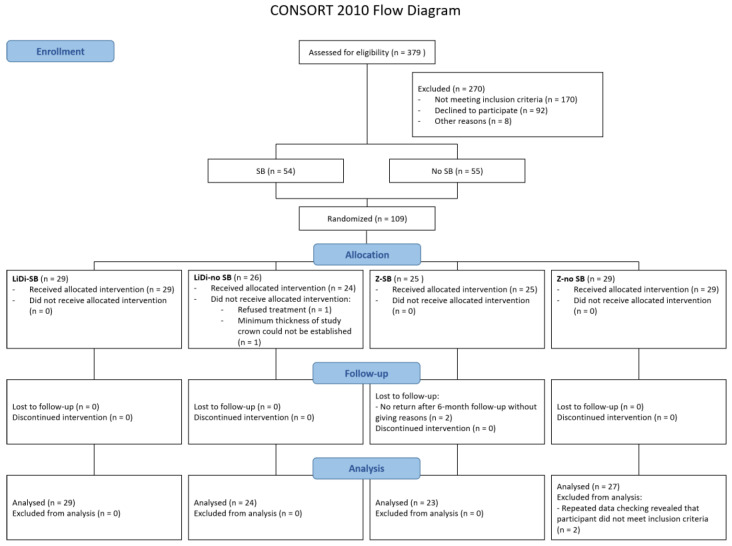
Participant flow diagram. LiDi: lithium disilicate, Z: zirconia, SB: sleep bruxism.

A total of 379 patients were screened for eligibility. Of these, 109 patients were enrolled in the study and assigned to one of four study groups (Figure 1): LiDi-SB (*n* = 29), LiDi-no SB (*n* = 26), Z-SB (*n* = 25), and Z-no SB (*n* = 29). Six female participants were excluded from the one-year analysis (two each from the LiDi-no SB, Z-SB, and Z-no SB groups) for various reasons (Figure 1).

The characteristics of the analyzed participants and their SCs are shown in Table 1.

**Figure 2:**
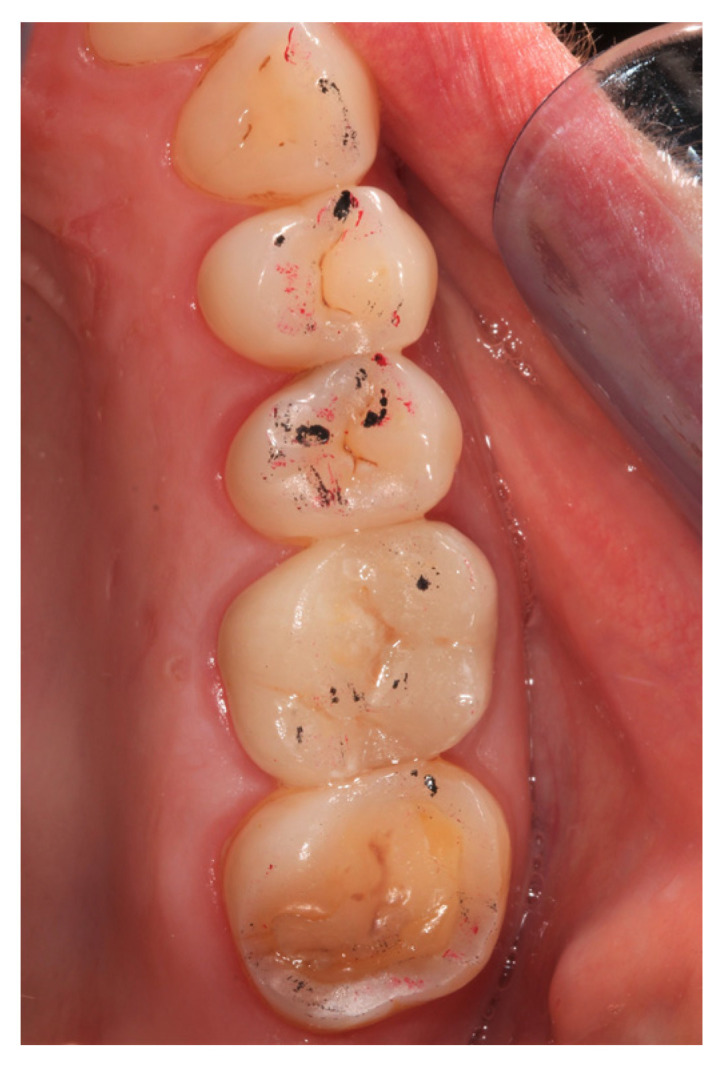
Participant with first upper left molar restored by study crown made of lithium disilicate. Static occlusion contacts were visualized using black occlusion foil, dynamic contacts using red occlusion foil.

**Figure 3:**
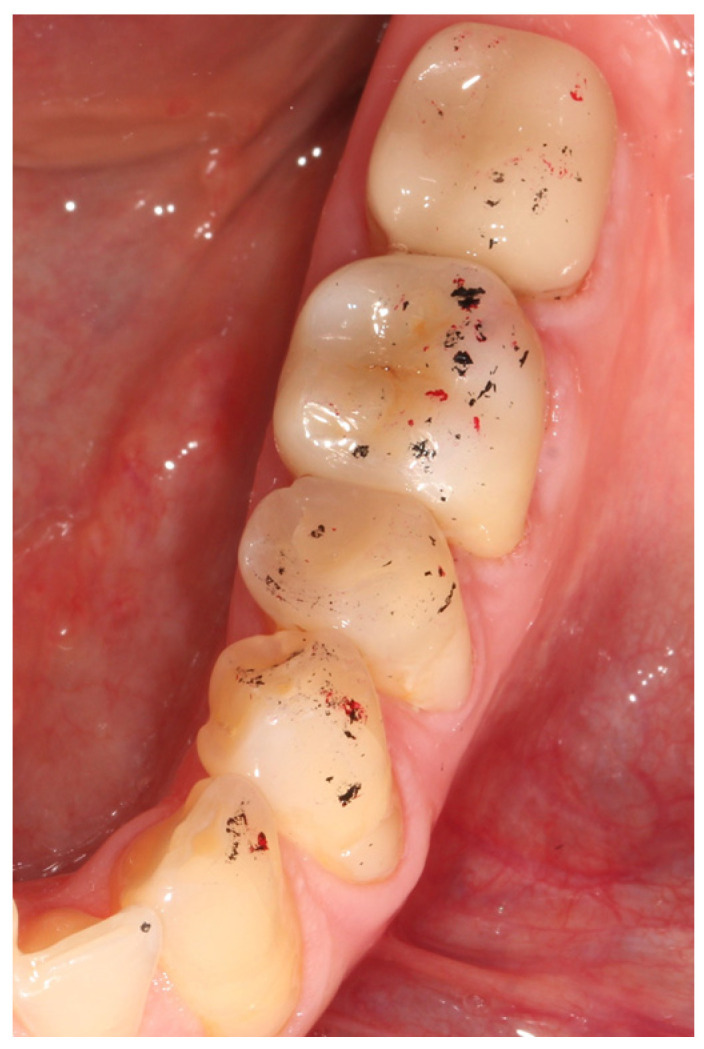
Participant with first lower left molar restored by study crown made of zirconia. Static occlusion contacts were visualized using black occlusion foil, dynamic contacts using red occlusion foil.

Examples of the LiDi and Z SCs are shown in Figure 2 and Figure 3.

### 3.2. Complications and Failures

The events recorded for the study crowns and the respective abutment teeth are listed in Table 2. There were no ceramic defects. The most common complication was symptomatic irreversible pulpitis of the abutment tooth, which was observed in three participants, one each from the LiDi-SB (3.4%), LiDi-no SB (4.2%), and Z-SB (4.3%) groups. In all affected participants, pulpitis was successfully handled with endodontic treatment performed through the crown in situ and the SC was preserved. One participant (4.3%) from the Z-SB group was diagnosed with a vertical root fracture of the abutment tooth, and the affected tooth was removed. The intact SC was removed from one participant (3.7%) in the Z-no SB group because the tooth had to be used as an abutment for a fixed partial denture after an adjacent tooth was lost.

### 3.3. Technical Complication, Survival, and Success Rates

The rate of technical complications was 0% in all study groups. The restoration survival rate was 100% in the LiDi-SB (29/29) and LiDi-no SB (24/24) groups, 95.7% (22/23) in the Z-SB group, and 96.3% (26/27) in the Z-no SB group (95% CI difference in proportions (DIP) -0.12 to 0.1, *p* > 0.999). Success rates were 96.6% (28/29) in the LiDi-SB group, 95.8% (23/24) in the LiDi-no SB group (95% CI DIP -0.097 to 0.11, *p* > 0.999), 91.3% (21/23) in the Z-SB group, and 96.3% (26/27) in the Z-no SB group (95% CI DIP -0.19 to 0.085, *p* = 0.588).

## 4. Discussion

In 2018, a systematic review assessed the influence of SB on the complication rate of tooth-supported ceramic restorations [6]. Eight studies were identified and included in the qualitative synthesis—three of these had a moderate risk of bias, and five had a high risk of bias. Furthermore, the overall quality of evidence in the included studies was very low according to GRADE criteria. The authors concluded that an association between SB and the failure of ceramic restorations could not be proven based on the current evidence. This finding is in accordance with the results of the present study.

Since this 2018 systematic review was published, some additional clinical trials and reports have assessed the association between SB and the failure of ceramic restorations [20,21,22]. These studies found no evidence that SB is a severe risk factor for restoration failure; however, assessing the association between SB and failures in all ceramic restorations is challenging because certain criteria need to be met. SB must be diagnosed using valid and reliable instruments; inclusion criteria must be strict; the study design should be a randomized, prospective clinical trial; and restorations should be made in a standardized way. The identification of bruxers and non-bruxers is particularly demanding, and numerous studies have failed to use valid protocols, which has resulted in severe bias [23]. Some studies have also assessed the influence of SB on veneered ceramics instead of monolithic ceramics [24].

In 2019, a study of 95 posterior monolithic Z units in 45 patients showed that 80% of catastrophic failures occurred in patients with clinical signs of bruxism [25]. However, this study included both tooth-supported (*n* = 10) and implant-supported (*n* = 85) restorations, and SB was diagnosed based on clinical signs only; therefore, these results cannot be compared with the results of the present study.

In a randomized clinical trial of monolithic Z and LiDi SCs [26], no fractures or chip-off fractures were observed after three years. The authors concluded, that “ceramic crowns in the posterior dentition made of monolithic translucent ZrO_2_ and LDS [authors’ note, lithium disilicate] show equal and promising clinical results from a short-term perspective.” This finding is in accordance with the findings of the present study. However, the present study also showed that not only the restoration material but also SB did not affect restoration success, whereas Gardell et al. did not diagnose SB nor include SB in the analysis.

Rauch et al. assessed the ten-year survival of chairside-generated monolithic LiDi SCs in 34 patients [27]. Two technical complications were observed: one molar SC needed to be recemented, and, after 2.8 years, and one molar SC was fractured. Bruxers were not excluded, so it is assumed that some bruxers took part. However, SB was not clinically assessed so the observed complications cannot be attributed to SB.

In the present study, no technical complications (ceramic complications/fractures) were observed in the restorations, so further recruitment was terminated according to the two-stage study protocol. Recent studies have provided evidence for low fracture rates in monolithic LiDi and Z SCs. In a 7.5-year laboratory survey, fracture rates were 262/27346 (0.96%) for LiDi SCs and 416/77411 (0.54%) for Z SCs [28]. In clinical trials on posterior monolithic LiDi SCs (850 restorations), fracture rates of 0% to 3.2% were detected after a mean observation period of 25.5 to 121.2 months [26,27,29,30,31]. Low fracture rates were also reported for monolithic LiDi SCs placed in posterior teeth [32], where 16/1782 restorations fractured over 16.9 years (annual fracture rate, 0.16%). Similar data have been reported for monolithic Z SCs. In a 2021 systematic review and meta-analysis, the fracture rate of 1657 Z SCs (88.9% posterior SCs) was 0.18% for a mean time of 1.07 years [33]. Even though no technical defects were found on the crowns in this study, it cannot be ruled out that changes have occurred at the microscopic level. For example, a recently published study showed that under cyclic loading there was a reduction in marginal adaptation in adhesively luted molar crowns made of materials similar to those used here; however, the resulting marginal gaps were within the clinically acceptable range [34].

The restoration survival and overall complication rates observed in the present study were similar to those reported in other studies on LiDi and Z SCs. In a recent review of 17 studies, the clinical performance of tooth-supported LiDi SCs (*n* = 2120) and Z SCs (*n* = 316) was assessed [35]. For the predominantly monolithic LiDi SCs, survival rates ranged from 83.5% to 100%, and complication-free survival rates from 71.0% to 96.7% after a mean follow-up time of 25.5 to 121.2 months. For Z SCs, the survival rates were 82.0% to 100%, and the complication-free survival rates were 64.0% to 100% after a mean follow-up period of 25.3 to 49.0 months. However, only veneered Z SCs were evaluated. Data on the survival rates of monolithic Z SCs were reported in the above-mentioned systematic review and meta-analysis [33]. Here, the survival rates of the 1657 restorations ranged from 91% to 100% for mean follow-up periods of 0.3 to 2.1 years. In a more recent study, a survival rate of 93.1% for 86 monolithic Z SCs (96.5% of which were posterior) was observed after a mean observation period of 6.3 years [36].

The present study also investigated non-ceramic complications and found no differences with the results of other studies. Abutment tooth fractures, root fractures, caries, tooth extractions due to periodontal or endodontic problems, hypersensitivities, endodontic treatments, loss of retention, and crown removals due to prosthetic reprovision were all documented in LiDi and Z SCs restorations, in agreement with previous findings [33,35].

This study has some limitations. First, polysomnography is the gold standard technique for assessing SB [7,37], but here electromyography with the BruxOff device was used. However, the validity of the BruxOff device was investigated in 25 participants and showed a high correlation with polysomnography (Pearson`s r = 0.95) [11]. Thus, the BruxOff device was deemed a valid and reliable method for diagnosing SB. Second, signs of SB might fluctuate in some individuals over time [38], so it is possible that some participants were deemed non-bruxers after the initial assessment but then showed signs of bruxism during the study. However, the technical failure rate did not differ between bruxers and non-bruxers, so this effect is probably negligible. Third, because published data are lacking, the sample size was calculated based on a pilot phase of the study (stage I), which included 50 participants. Thus, the sample size was based on convenience and was not data-driven. However, data of the same level of evidence have not yet been published, so the present results are still valuable. Fourth, the results presented are from one year of observation and a longer observation would be desirable.

## 5. Conclusions

Within the limitations of this study, no short-term influence of SB was demonstrated on the technical complication rates, survival rates, and success rates of both LiDi and Z SCs on natural teeth.

## Figures and Tables

**Table 1:** Participant and restoration characteristics in study groups. LiDi: lithium disilicate, Z: zirconia, SC: single crown, SD: standard deviation, SB: sleep bruxism, PPD: probing pocket depth.

Characteristic	Outcome	LiDi-SB *n* = 29	LiDi-no SB *n* = 24	Z-SB *n* = 23	Z-no SB *n* = 27
Sex, n (%)	Man	10 (34.5)	10 (41.7)	15 (65.2)	8 (29.6)
	Woman	19 (65.5)	14 (58.3)	8 (34.8)	19 (70.4)
Participant age at SC cementation, mean (SD)		48.1 (12.21)	56.2 (12.33)	48.7 (10.49)	62.0 (8.44)
SB categories, n (%)	Moderate SB	15 (51.7)	0 (0)	7 (30.4)	0 (0)
	Severe SB	14 (48.3)	0 (0)	16 (69.6)	0 (0)
	No SB	0 (0)	24 (100.0)	0 (0)	27 (100.0)
Tooth, n (%)	1st molar	21 (72.4)	16 (66.7)	20 (87.0)	16 (59.3)
	2nd molar	8 (27.6)	8 (33.3)	3 (13.0)	11 (40.7)
Jaw, n (%)	Maxilla	8 (27.6)	8 (33.3)	9 (39.1)	7 (25.9)
	Mandible	21 (72.4)	16 (66.7)	14 (60.9)	20 (74.1)
Endodontic status, n (%)	Vital	22 (75.9)	19 (79.2)	15 (65.2)	21 (77.8)
	Endodontically treated	7 (24.1)	5 (20.8)	8 (34.8)	6 (22.2)
PPD (mm, deepest of 6 measurements per tooth), mean (SD)		3.2 (0.62)	3.0 (0.62)	2.8 (0.58)	3.0 (0.55)
PPD antagonist (mm, deepest of 6 measurements per tooth), mean (SD)		3.1 (0.86)	3.1 (0.93)	3.1 (0.60)	3.3 (1.12)
Tooth mobility (0–3) ^1^, n (%)	0	29 (100.0)	23 (95.8)	23 (100.0)	27 (100.0)
	1	0 (0)	1 (4.2)	0 (0)	0 (0)
Tooth mobility antagonist (0–3) ^1^, n (%)	0	29 (100.0)	24 (100.0)	23 (100.0)	26 (100.0)
Missing teeth ^2^, n (%)	0	23 (79.3)	22 (91.7)	20 (87.0)	19 (70.4)
	1	4 (13.8)	2 (8.3)	3 (13.0)	5 (18.5)
	2	2 (6.9)	0 (0)	0 (0)	3 (11.1)
Occlusal guidance	Canine	9 (31.0)	6 (25.0)	6 (26.1)	6 (23.1)
	Group	20 (69.0)	18 (75.0)	17 (73.9)	20 (76.9)
Minimum occlusal SC thickness (mm), mean (SD)		1.4 (0.17)	1.4 (0.14)	1.3 (0.13)	1.4 (0.15)
Minimum axial wall thickness (mm), mean (SD)		1.2 (0.13)	1.2 (0.14)	1.2 (0.15)	1.2 (0.17)
Occlusal adjustments prior to SC cementation, n (%)	Yes	22 (75.9)	22 (91.7)	19 (82.6)	22 (84.6)
	No	7 (24.1)	2 (8.3)	4 (17.4)	4 (15.4)
Occlusal adjustments after SC cementation, n (%)	No	23 (79.3)	20 (83.3)	23 (100.0)	23 (85.2)
	Yes, one	6 (20.7)	4 (16.7)	0 (0)	4 (14.8)
	Yes, multiple	0 (0)	0 (0)	0 (0)	0 (0)

^1^ Categories not occupied, not shown for clarity. ^2^ Missing posterior teeth that have neither been replaced prosthetically (fixed prosthesis) nor treated orthodontically (gap closure).

**Table 2:** Tooth- and restoration-related events in study groups. LiDi: lithium disilicate, Z: zirconia, SC: single crown, SB: sleep bruxism.

Event	LiDi-SB *n* = 29	LiDi-no SB *n* = 24	Z-SB *n* = 23	Z-no SB *n* = 27
Technical complications/ceramic defects, n (%)	0 (0)	0 (0)	0 (0)	0 (0)
Symptomatic irreversible pulpitis, n (%)	1 (3.4)	1 (4.2)	1 (4.3)	0 (0)
Vertical root fracture, n (%)	0 (0)	0 (0)	1 (4.3) ^1^	0 (0)
Intact SC removed, n (%)	0 (0)	0 (0)	0 (0)	1 (3.7) ^1^

^1^ Classified as failure.

## Data Availability

The data underlying the results of this study can be requested from the corresponding author. This requires a justified interest.
